# Assessing the number of ancestral alternatively spliced exons in the human genome

**DOI:** 10.1186/1471-2164-7-273

**Published:** 2006-10-24

**Authors:** Rotem Sorek, Gideon Dror, Ron Shamir

**Affiliations:** 1 Department of Human Genetics, Sackler Faculty of Medicine, Tel Aviv University, Tel Aviv 69978, Israel; 2 The Academic College of Tel-Aviv-Yaffo, Tel-Aviv, 64044, Israel; 3 School of Computer Science, Tel Aviv University, Tel-Aviv 69073, Israel; 4 Genomics Division, One Cyclotron Road, MS 84-171, Lawrence Berkeley National Laboratory, Berkeley, CA 94720, USA

## Abstract

**Background:**

It is estimated that between 35% and 74% of all human genes undergo alternative splicing. However, as a gene that undergoes alternative splicing can have between one and dozens of alternative exons, the number of alternatively spliced genes by itself is not informative enough. An additional parameter, which was not addressed so far, is therefore the number of human exons that undergo alternative splicing. We have previously described an accurate machine-learning method allowing the detection of conserved alternatively spliced exons without using ESTs, which relies on specific features of the exon and its genomic vicinity that distinguish alternatively spliced exons from constitutive ones.

**Results:**

In this study we use the above-described approach to calculate that 7.2% (± 1.1%) of all human exons that are conserved in mouse are alternatively spliced in both species.

**Conclusion:**

This number is the first estimation for the extent of ancestral alternatively spliced exons in the human genome.

## Background

In recent years, numerous studies have shown that alternative splicing is very prevalent in the human genome. Assessing the number of genes that undergo alternative splicing has drawn much attention since the emergence of ESTs as a resource for global alternative splicing analysis. Early studies estimated this number at 35% [1] 38% [2] and 42% [3] while recent studies present numbers as high as 59% [4] 74% [5] and even higher [6,7]. However, there is an ongoing debate as to how many of these predicted splice variants are functional, and how many are the result of aberrant splicing (or 'noise') [8,9]. In addition, ESTs provide only a sample of the human transcriptome, and many splice variants (including evolutionarily conserved ones) are not represented in public EST databases. The number of human genes that undergo alternative splicing, therefore, remains elusive.

But does this number reveal the full picture of the transcriptome complexity? Some genes can produce extremely high number of transcripts, while others produce only two or three variants. For example, the drosophila DSCAM gene, which harbors 95 alternatively spliced exons, can theoretically produce up to 38,016 different transcripts. This number of transcripts, produced from this single gene, is twice the number of genes in the entire fly genome. Therefore, the number of **exons **that undergo alternative splicing is a crucial parameter, and gives new, complementary information that is not contained in the number of alternatively spliced **genes**. As far as we are aware, the question of assessing the number of alternative exons was never addressed in the literature before.

We have recently described an accurate machine-learning method allowing the detection of conserved alternatively spliced exons without using ESTs [10,11]. This approach relies on specific features of the exon and its genomic vicinity that distinguish alternatively spliced exons from constitutive ones. Since this method does not rely on ESTs, it avoids the problems of sampling errors and noise inherent to ESTs, and can therefore provide robust assessment on the absolute number of conserved alternatively spliced exons in the human genome.

## Results

### Classifying ancestral alternative exons

Human internal exons (excluding the first and last ones) can roughly be divided into three categories, based on comparison to their counterparts in mouse: (i) **constitutive exons**, i.e., exons that occur in the same form in all (one or more) splice variants of the gene they belong to; (ii) **ancestral alternative exons**, which are exons that are alternatively spliced both in human and in mouse, i.e., were alternative in the common ancestor of primates and rodents, and remained so in both species; (iii) **human-specific alternative exons**, which are exons that have alternative splicing pattern in human but not in mouse [12].

To distinguish between ancestral alternative exons and other exons we used a classifier trained by Support Vector Machine (SVM), a machine learning algorithm [11]. For each exon, the features taken into account were (i) exon length, (ii) exon length divisibility by three, (iii) percent identity when aligned to the mouse counterpart, (iv) conservation in the upstream and downstream flanking intronic sequences (4 features), (v) 5' splice-site composition (28 features), (vi) poly-pyrimidine tract intensity, and (vii) 3-tuple counts in the exon and the flanking introns (192 features). These features were all previously shown to differ between alternative and constitutive exons [10,11,13,14]. For detailed description of our classifier see ref [11].

To obtain an accurate classifier we first collected pre-calculated training sets of each of the three groups of exons: (i) 5069 constitutive exons from Yeo et al [13]; (ii) 241 ancestral alternative exons compiled by the same authors [13] and (iii) 212 human-specific alternative exons compiled by Pan et al. [12]. After filtering (see Methods), the final sets contained 4655 constitutive exons, 221 ancestral alternative exons, and 182 human specific alternative exons. We also used a second set of 243 (215 after filtering) ancestral alternative exons compiled in [15] as an independent training set.

**Figure 1:**
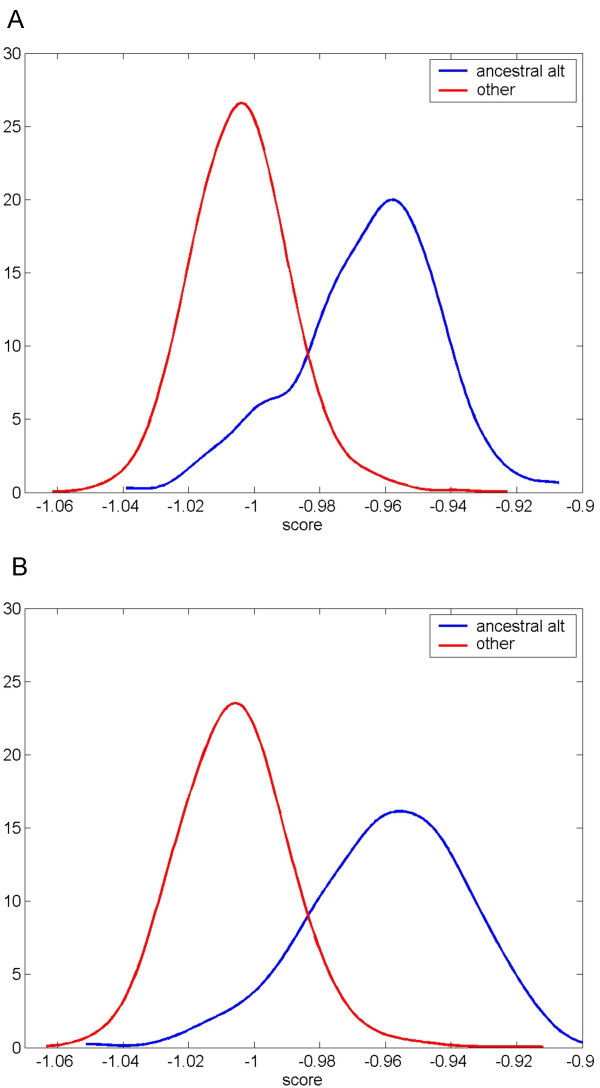
Score distribution of ancestral alternative exons and "other" exons. X axis: the SVM 'alternativity score'. Y Axis: probability density. 'ancestral alt', score distribution of ancestral alternative exons in the training set; 'other', score distribution of 4655 constitutive exons plus 182 human specific alternative exons in the training set. (A) Model trained with 221 ancestral alternative exons from Yeo et al. [13]. (B) Model trained with 215 ancestral alternative exons from Sorek et al. [15].

Next, we trained the classifier to distinguish between the ancestral alternative exons (221 exons) and the **'other' **exons, i.e., the combined group of constitutive and human-specific alternative (4655+182 = 4837) exons. For each exon, the trained classifier outputs an 'alternativity score', a number that is indicative to whether that exon is ancestrally alternative or not. Using ten-fold cross validation we estimated the distributions of these scores for alternative and other exons. Figure 1 presents these score distributions. As seen in the figure, the scores significantly distinguish ancestral alternative exons from the other exons. It is noteworthy that we could not find a set of parameters that distinguishes well between the constitutive and the human-specific alternative exons, indicating that these two sets of exons have very similar characteristics.

### Genome-wide collection of human exons

To assess the fraction of conserved, ancestral alternative exons out of the entire human exon population, we first used ESTs and cDNAs from Genbank version 136 aligned to the human genome (version hg16) to identify internal human exons. We collected 120,964 internal exons for which we were able to identify the mouse ortholog exon as described in ref [10]. These exons constitute the set of "conserved exons". This set of exons is the basis for our analysis, as our classifier relies on human-mouse homology-based features.

### Fraction of ancestral alternative cassette exons

**Figure 2:**
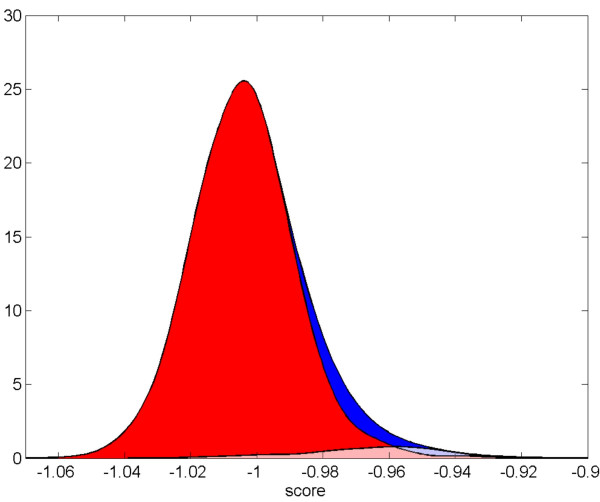
Decomposition of score distribution of 120,964 human-mouse conserved exons. The blue distribution represents the scores of all exons. Its bigger right tail represents the contribution of the ancestral alternative exons. This distribution was decomposed (see text) into "ancestral alternative" (transparent) and 'other' (superimposed in red) distributions. The area under the "ancestral alt" curve represents the percentage of ancestral alternative exons out of the entire exons set.

Next, we employed our classifier on the entire set of 120,964 conserved exons (Figure 2). The resulting score distribution represents a combination between "ancestral alternative" and "other" exon scores, with the observed right tail clearly representing the contribution of ancestral alternative exons (Figure 2). To decompose the distribution into its two components we used a simple mixture model: Assume that the distributions of scores of "ancestral alternative" and "other" exons are represented by the functions *f*_*a*_(*x*) and *f*_*o*_(*x*) respectively. Then the distribution of the entire population of 120,964 exons composed of these two components is modeled by *f*(*x*) = *p f*_*a*_(*x*) + (1-*p*) *f*_*o*_(*x*), where *p *is the relative fraction of ancestral alternative exons. Estimating the three distributions *f*_*a*_, *f*_*o *_and *f *using standard Maximum Likelihood estimation (see Methods), we calculated that *p *equals 3.6% (± 0.5%).

To check the possibility that this estimator was biased by the training set of ancestral alternative exons, we repeated our analysis using the second training set of 215 ancestral alternative exons from Sorek et al [15]. This exon set overlaps the Yeo et al set by only 38 exons, and can therefore be used to obtain another estimator that is to a large extent independent from the former. We trained a second model on the basis of this second set of exons versus the set of 4837 other exons (Figure 1B), and employed the resulting model on the entire set of 120,964 conserved exons. After decomposing the score distribution, the resulting fraction of ancestral alternative exons was 3.9% (± 0.5%), very similar to the result obtained using the first training set. Combining these two results, we conclude that 3.75% (± 0.65%) of the exons in our set of 120,964 known human-mouse conserved exons are ancestral alternative cassette exons (Figure 2).

### The hidden conserved alternative exons

Our method enables, for each known exon, to assess its probability of being alternatively spliced. However, some conserved internal alternatively spliced exons are not represented in EST data due to sampling limitations of ESTs. How many such exons still await discovery? To assess their number we used the output of the Exoniphy program [16]. This program, designed by Siepel and Haussler, identifies evolutionarily conserved protein-coding exons using a phylogenetic hidden Markov model, which simultaneously describes exon structure and exon evolution [16]. Using human mouse and rat genome alignment, Exoniphy predicted 177,040 putative exons on the UCSC hg16 genome version (Methods). As these predictions do not depend on existence of ESTs, they can be used to assess the number of conserved coding exons that are still undetected.

Out of our set of 120,964 conserved exons, 105,740 (87%) are accurately predicted by Exoniphy. This indicates the small amount of false negative predictions, namely, most true conserved exons are predicted by Exoniphy. To search for possible new alternatively spliced exons in the remaining 71,300 Exoniphy predictions, we took all such predictions that satisfy the following criteria: (i) Found within an intron of a known RefSeq gene (ii) Do not overlap with any EST/RNA, (iii) Do not overlap with any processed pseudogene, and (iv) Are flanked by canonical splice sites both in the human and the mouse genomes. The first two criteria screened the vast majority of the remaining predictions, as most of the predicted exons were either overlapped (fully or partially) by an expressed sequence, or not contained within any known intron.

**Figure 3:**
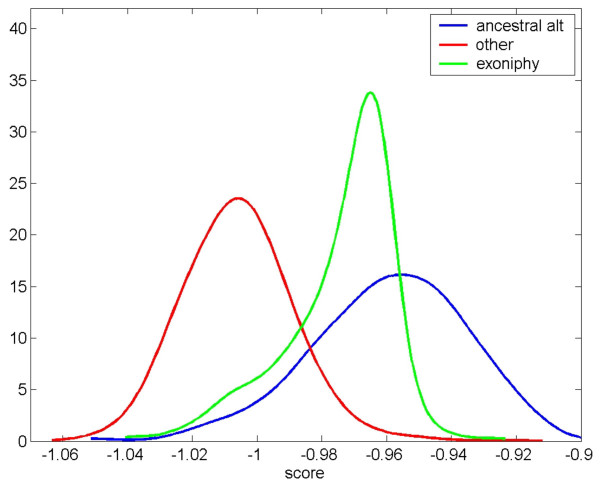
Score distribution for the exons predicted by 'Exoniphy'. 'ancestral alt', score distribution of ancestral alternative exons in the training set; 'other', score distribution of 4655 constitutive exons plus 182 human specific alternative exons in the training set; 'exoniphy', scores of 783 predicted Exoniphy exons. The distribution of the Exoniphy exons is clearly biased toward the 'ancestral alt' distribution, indicating that many of the Exoniphy exons are truly ancestral alternative exons.

We collected 783 putative novel exons conforming to the above criteria, and applied our classifiers on each of these. Figure 3 presents the 'alternativity scores' distribution of the 783 putative exons. Indeed, it is apparent that this population of sequences is highly enriched in true alternative exons. Using the criterion described above to decompose the distribution, we calculated that 93.1% (± 3.7%) of these exons (~730 exons) represent true cassette exons. These results are in agreement with recent estimates that few hundred ancestral alternative exons are still undetected in the human genome [17].

Our analysis indicates that hidden, novel exons contribute at least 0.6% (out of ~122,000 conserved exons) to the overall fraction of ancestral alternative exons. Combined with our result from the non-hidden conserved exons, we estimate that 4.3% (± 0.65%) of all conserved exons in human are ancestral alternatively spliced cassette exons. We note that Exoniphy is expected to fail in the identification of very short and highly conserved exons (A. Siepel, personal communications). As alternative exons tend to be smaller and more conserved than constitutive ones [10] the actual numbers of 'hidden' alternative exons might be higher than that we calculate.

### Additional types of alternative splicing

Both our training sets are composed solely of ancestral alternatively spliced exons of the "cassette" type, i.e., such that undergo exon-skipping. Exon skipping is the most abundant type in human, composing ~40% of all alternative splicing events in the genome [18]. Events such as mutually exclusive exons, intron-retention, alternative 5' and 3' splice sites, etc., comprise the remaining 60% of the events. Due to the composition of the training sets, we expected our classifiers to be biased towards identifying mainly cassette (skipped) exons. Indeed, manually inspecting EST support for 50 of the conserved exons that had the highest alternativity scores indicates that 70% of them are alternatively spliced in an exon-skipping manner, while only 30% show other patterns of alternative splicing. A similar 70:30 ratio was observed in the results obtained by using another classification rule, and also in the experimentally verified predictions of that rule [10]. Hence, exon-skipping cases are over-represented in our predictions, and consist a fraction of 0.7 of our set instead of the expected 0.4 [18].

To reach the total fraction of exons in the genome that undergo all types of alternative splicing, one must, therefore, multiply our previous results by a factor of 0.7/0.4 = 1.75 (this applies only to the known, non-hidden exons, as all the hidden exons are, by definition, of the cassette exons type). Thus, our estimate for the fraction of exons that are ancestrally alternatively spliced is 3.75% * 1.75 + 0.6% = 7.2%. The error in this estimate is at least ± 1.1% (0.65*1.75) due to the original error estimate; additional unknown error in the 1.75 factor may increase the error bound.

## Discussion

Our analysis indicates that 7.2% (± 1.1%) of the exons in the human genome are ancestrally alternatively spliced, i.e., undergo alternative splicing both in human and in mouse. As our positive training set was composed solely of ancestral alternative exons, the classifier cannot detect species-specific alternatively spliced exons. However, some alternative splicing events are species specific: For example, the primate-specific Alu-derived exon 8 in the RNA-editing enzyme ADAR2 is contained in 40% of all ADAR2 transcripts in human, thus changing the RNA-editing activity of the mature protein [19]. Moreover, EST analyses have shown that species-specific alternative splicing might be up to 3 or 4 times more abundant than conserved alternative splicing [10,20,21]. In this light, the total number of human exons that are alternatively spliced might be significantly higher than the 7.2% we calculated. However, many lines of evidence suggest that the majority of apparently species-specific alternative splicing events observed in dbEST represent non-functional alternative splicing [9,13]. As there are currently no computational methods to distinguish functional from non-functional species-specific splicing, at this stage it is impossible to determine the contribution of species-specific alternative splicing to the fraction of functional alternative exons.

Based on the calculated fraction of 7.2% (± 1.1%), we estimate that 7,500–10,000 exons in the set of ~121,700 conserved human exons (including the putative hidden exons) are ancestral alternatively spliced. This is a surprisingly small number; however, we note that our set of conserved exons is not the complete exon repertoire in the human genome, which was estimated to contain ~200,000 exons [22]. Presumably, fast-evolving exons (for example, in immune- or reproduction-related genes [22]) escape our set due to low human-mouse conservation.

Nevertheless, even if we assume that the same rate of alternative splicing that we found holds for the complete set of ~200,000 exons in human (distributed within ~25,000 genes), our results predict that the number of ancestral alternative exons in human is somewhere between 12,000–16,500. At first glance this number seems to contradict the current paradigm of alternative splicing abundance in human genes (the higher estimates are that 74% of all human genes are alternatively spliced [5]). Indeed, even if each alternatively spliced gene contains only one ancestral alternative exon (which is frequently untrue), the fraction of such genes would still be 48%-66%, less than 74% of all human genes. However, all high estimates of alternatively spliced genes were derived from analyses that took into account also species-specific splicing events, which, as mentioned above, are much more abundant than ancestral alternative splicing (and their functionality is yet to be proven). Our estimate solely refers to ancestral, non-species specific alternative exons.

Our estimation of the fraction of alternative exons is composed of three elements: estimation for the number of known exons that undergo conserved exon-skipping (3.75%), extrapolation using the fraction of the 'exon-skipping' type out of all types of alternative splicing (yielding a factor of 1.75), and additional estimation for the number of ancestral alternative exons still hidden in the human genome (0.6%). Whereas our results show that the basic estimate of 3.75% (± 0.65%) is robust, the two subsequent extrapolations are less reliable (due to the difficulty in assigning proper error bounds). These extrapolations should, therefore, be viewed with caution.

Several factors might have influenced our results. First, it is still possible that our training sets of exons are somehow biased and do not represent the actual qualities of alternative and constitutive exons in the genome. Although we are not aware of any reason for such bias, if it exists it could influence the resulting numbers. The fact that we obtained very close estimates using two different training sets, compiled in two separate studies by different methods, makes such bias less likely. Second, the number of 'hidden' alternative exons might be higher than what we detected, due to under-representation of very short and highly conserved exons in the Exoniphy predictions. Finally, some exons can be alternatively spliced in several ways, e.g., they can be both skipped and have an alternative 3' splice site. Our analysis, which assumed that ~40% of all alternative exons are of the exon-skipping type, did not take into account such complex cases.

It is noteworthy that our method does not classify each specific exon as ancestral alternative or 'other'. Instead, it assigns the exon a score that could be used to calculate the probability that it is ancestral alternative. Calculated over the entire set of conserved exons in the human genome, these probabilities enabled us to accurately estimate the number of ancestral alternative exons, but not to accurately pinpoint each one of them. For the same reason, the method cannot uncover the full complexity of the transcriptome, as it does not directly provide the number of alternative transcripts of each gene.

We could not find a set of parameters that produce SVM-based classifier that would distinguish between species-specific alternatively spliced exons and constitutive exons. This could stem from several factors. First, for the species-specific alternative exons we, naturally, did not have the comparative-genomics-based parameters (such as exon-conservation and flanking-intron conservation). As these are strong classifying parameters in our SVM classifier [11] their absence in this case limits its ability to distinguish between the two populations. Second, the other characteristics of species-specific alternative exons (such as their size distribution and divisibility by 3) resemble those of the constitutive exons [9].

## Conclusion

To our knowledge, this report contains the first estimation for the number of ancestral alternatively spliced exons in the human genome. Most or all of these exons represent functional and highly important splicing events (otherwise they would not have been conserved in evolution over millions of years). In the quest to reveal the full complexity of the transcriptome, the central parameter will be the total number of human functional transcripts. While this number still remains unknown, our estimate is an important signpost towards revealing it.

## Methods

### Data

To compile the "conserved exons" set, internal human exons were identified by aligning ESTs and cDNAs from Genbank version 136 [23] to the human genome (version hg16) downloaded from UCSC genome browser [24]. Internal exons were considered as such if they had valid flanking 3' and 5' splice sites. The resulting exons were compared to the mouse genome as described in ref [10]. Classifying features were calculated for each of the exons as described in ref [11].

Training set exons were downloaded from refs [12,13,15] and searched for matches in the "conserved exons" set (120,964 exons) using BLASTn. Exons with no perfect match were filtered out of the training set.

Exoniphy exons were downloaded from the UCSC hg16 genome version. EST/cDNA coverage of Exoniphy exons was also derived from the UCSC annotation.

### SVM training and hyper-parameter selection

To identify the ancestral alternative exons from species-specific alternative and constitutive exons, an SVM classifier with a Gaussian kernel was trained using the datasets described above. Soft-margin SVM implemented in SVMlight [25] was used throughout the research.

SVM training involves fixing several hyper-parameters, the values of which has a crucial effect on the performance of the trained classifier. A grid search combined with five-fold cross validation was used to evaluate the performance over various values of the slack parameter *C*, the width the kernel σ and the number of features. The latter was controlled by standard filtering using Pearson correlation as an association measure between a feature and the target. The final model, obtained by optimizing the area under the ROC curve, used all available features and its hyper-parameters were *C *= 0.1 and σ = 1.4. The "alternativity score" of an exon is the output of the trained SVM classifier when applied to its feature vector (For details see ref [11]).

### Likelihood maximization

Using the trained SVM we obtained several distributions of alternativity scores: the scores distribution of the ancestral alternative exons, the scores distribution of the 'other' exons, the scores distribution of all human exons, and the scores distribution of Exoniphy exons. The latter two populations contain unknown fractions of ancestral alternative exons, hence the distribution of each one of them can be decomposed into two components: *f*(*x*) = *pf*_*a*_(x) + (1-*p*)*f*_o_(*x*) where *f*_*a *_and *f*_*o *_represent the probability distribution function of the SVM score *x *for the ancestral alternative exons and for the 'other' exons (ancestral constitutive and human-specific alternative), respectively. *p *is the fraction of ancestral alternative exons (0 ≤ p ≤ 1). *f *represents the distribution function of a mixed population, either all human exons or Exoniphy exons.

*p *was computed by maximizing the log likelihood function L = log Π_*i*_f(*x*_*i*_) = Σ_*i *_log(*pf*_*a*_(*x*_*i*_) + (1-*p*)*f*_*o*_(*x*_*i*_)), where the summation is over the SVM scores of all exons in the test set. As *L *is a function of a single parameter, finding its global maximum is straightforward. The Fisher information *F *was used to estimate a confidence interval for *p*. In subsequent analysis we used one standard deviation Δ = 1/√*F *as a measure of the uncertainty in *p*.

### Parzen smoothing

*f*_*a *_and *f*_o_were extracted from the score distribution of the ancestral alternative exons and the 'other' exons of the training set, respectively, using 200-fold cross validation to avoid over-fitting. The score distributions are only insignificantly different if a 'leave-one-out' cross-validation is performed instead.

Since a normal approximation of these distributions deviated considerably from the observed distributions, Parzen smoothing was used instead to model them as continuous probability distribution functions. To determine the Gaussian width for Parzen smoothing, each population, ancestral alternative exons and 'other' exons, was split into two equal disjoint sets. One set was used to produce a probability distribution function using a fixed Gaussian width, and the second set was used to calculate the log-likelihood function L_*a *_= Σ_*i *_log(*f*_*a*_(*x*_*i*_)) and L_o_= Σ_*i *_log(*f*_*o*_(*x*_*i*_)) for the ancestral alternative exons and 'other' exons, respectively, where the summation is over the examples of the second set. This process was repeated 100 time for each Gaussian width. The widths that gave the highest average log-likelihood values were chosen for Parzen smoothing. These widths were 0.0111 and 0.0117 for the ancestral alternative and the 'other' exons, respectively, in the Yeo set, and 0.0159 and 0.0145 for the ancestral alternative and the 'other' exons, respectively, in the Sorek set.

## Authors' contributions

R. Sorek participated in the study design and performed the data collection and analysis. GD participated in the study design and performed the SVM and statistical analyses. R Shamir participated in the design and coordination of the study. All authors read and approved the final manuscript.
